# Surgery of the Liver

**Published:** 1905-09-09

**Authors:** 


### SURGERY OF THE LIVER.

**Hepatic Drainage.**—Infection and disease of the ducts of the liver and gall-bladder have been studied with great minuteness by pathologists and surgeons, and Deaver[1] considers that the treatment of these conditions is beginning to rest on a sound and rational basis. The liver, the gall-bladder, and the biliary tract become infected by the ascent of bacteria from the duodenum by way of the common bile-duct, or by an infection of the liver from the blood in the portal vein. We may assume with Adama (1) that the colon bacilli in small numbers are, in the healthy individual, constantly finding their way into the finer branches of the portal circulation; and (2) that one of the functions of the liver is to arrest the further passage of these bacilli into the general circulation, and to destroy them through the agency of the specific cell of the organ. It is therefore quite apparent that when the specific action of the liver cells has been disabled by the toxic products of the bacteria which are invading the organ, the bacteria may reach the bile and spread through the gall-bladder and ducts, and recurring attacks of inflammation in the gall-bladder will gradually thicken its walls and lead to shrinkage of the organ, with, perhaps, strictures or contortions. Cirrhosis of the liver may be the direct result of infection ascending the common duct from the duodenum, or through the portal circulation from the intestine. The gall-bladder must be recognised as the source of infection not only in hepatitis and pancreatitis, but also in 90 per cent. of all the cases of common duct disease, and operation should be performed before the infection and gall-stones invade and damage the common duct. As a result of his wide experience Deaver considers that in complicated as well as uncomplicated cases, operation should be resorted to as soon as it is definitely known that gall-stones are present.

Cholecystostomy is the operation of choice in the majority of lesions of the biliary tract. It is indicated in most of the inflammatory affections of the gall-bladder itself. The liver may be drained of infectious bile without injury of its passages, and with the ability to restore the function of the gall-bladder. When the common duct is obstructed by inflammatory swelling of its own lumen, or of the head of the pancreas, cholecystostomy relieves the pressure of bile and places the inflamed parts in a condition of rest. The operation is indicated in (1) very acute infection of the gall-bladder; (2) whenever cysticotomy or choledochotomy is performed, except when cholecystectomy is also done; (3) in chronic conditions, except when cholecystectomy is indicated; (4) in chronic pancreatitis. Acute infections of the gall-bladder practically always presuppose infection of the common and hepatic ducts, probably also of the liver; and, as drainage is essential, the gall-bladder must be preserved if possible. While the liver can be drained by tubage of the common or hepatic ducts, such drainage is attended by more risk and is less likely to be complete than drainage through the gall-bladder. The practice of simple drainage of the gall-bladder in certain cases of empyema with involvement of the surrounding structures fearing the dissemination of sepsis, is irrational; as, for example, where the cystic duct is occluded by a stone, the removal of which, and the establishment of bile drainage is essential for a favourable outcome of the case. In the performance of cholecystostomy Deaver does not stitch the gall-bladder to the peritoneum and aponeurosis, but fastens a tube into the opening in the gall-bladder without fixation of the latter. The only exception to be made to this method is in the class of infectious cases where prolonged drainage is required. In these cases it is better to adopt the old method of sewing the gall-bladder to the aponeurosis as the opening keeps patulous for a longer period.

Cholecystectomy is indicated (1) in chronic obstruction of the cystic duct due to a stricture following old ulceration, or to impaction of a calculus within the spiral folds of the mucous membrane, and producing hydrops of the gall-bladder; (2) in chronic calculous cholecystitis of a shrunken, functionless gall-bladder with thickened walls, provided the common duct is free and there have been no clinical indications of existing infection; (3) in empyema of the gall-bladder, when the organ is seriously damaged; (4) in gangrene of the gall-bladder; (5) in carcinoma of the gall-bladder. This, however, leads us to the vexed question of the desirability of removing the gall-bladder as a routine measure. Against this Brownlee[2] raises a protest. In his opinion the results of ruthless excision of the gall-bladder are not evident immediately after operation, and will be proved only by a gradual development of evils beginning at this time. He maintains that when there is no gall-bladder into which the bile may for a time be forced, the bile is at once backed up into the liver, causing an acute hepatitis which interferes with its normal function, and a double process begins at once. The organ ceases to remove the bile from the blood, or does so only in part; that removed begins immediately to be re-absorbed, and the patient is rapidly overwhelmed with sepsis. If the cystic duct is closed permanently the bladder is of course useless, and should be removed. The same indications exist when the gall-bladder has become permanently diseased from any cause, but there is no justifiable reason for removing every gall-bladder which has become infected, or has afforded lodgment for stones. The mere fact that a man can live without it is not sufficient. We are not at all sure that his liver functions are not materially embarrassed, which in the end will aid in the downfall of his general health. Brownlee doubts if the number of recurrences of stones in the gall-bladder after cystotomy is greater than the number of cases of stones lodged in the common duct after the bladder has been removed, and the danger of reinfection is less. It is a fact that infection of the gall-bladder a second time is an exception to the prevailing rule, as is also a second accumulation of stones; whether an immunity is developed, or an insufficient number of years have elapsed to prove the frequency of second accumulations one cannot say. Cholecystectomy is still too new for us to predict what will follow, but it is probable that stones will form more readily in a distended common duct where the gall-bladder is absent, and that infection leading direct to the liver will have easier access, which, with the evil of the liver having to store a quantity of its own production within itself for variable lengths of time, will ultimately lead to the downfall of the patient's health.

[1] Brit. Med. Jour., Oct. 1, 1904. [2] Med. Rec., Dec. 10, 1904, p. 933.

